# Influence of generic reference pricing on medicine cost in Slovenia: a retrospective study

**DOI:** 10.3325/cmj.2018.59.79

**Published:** 2018-04

**Authors:** Nika Marđetko, Mitja Kos

**Affiliations:** Chair of Social Pharmacy, University of Ljubljana, Faculty of Pharmacy, Ljubljana, Slovenia

## Abstract

**Aim:**

To assess the impact of the generic reference pricing (GRP) system on the prices and cost of medicines in Slovenia approximately 8 years after its introduction in 2003 and before the implementation of the therapeutic reference pricing system.

**Methods:**

A retrospective study of all medicines (N = 789) included in the GRP system on January 31, 2012 was performed. Medicine prices and cost were analyzed between January 31, 2012 and December 31, 2013 after every update (N = 11) of the maximum reimbursable price (MRP) and were compared to the price and cost on January 31, 2012 (index date). Time trends of different types of medicine prices (maximum allowed price, MRP, and actual wholesale price) were graphically analyzed, and actual wholesale price adjustments to the MRP changes and the budget impact of the GRP were assessed.

**Results:**

In the 2-year study period, the long-term performance of the GRP system was associated with an approximate 45% decrease in the average MRP or an approximate 20% cost reduction. For each MRP update period, the GRP reduced the cost based on the maximum allowed price for approximately 30%. The wholesale price adjustments were mostly made for medicines priced above the MRP and reduced patients’ out-of-pocket cost.

**Conclusions:**

In the long term, the GRP system effectively reduced medicine prices and the cost of reimbursed products.

Similar to other European countries, Slovenia has been challenged by increasing pharmaceutical expenditures (1). In 2013, the total pharmaceutical expenditure in Slovenia accounted for 20.4% of the total health care spending. In terms of the public payer expenditure, prescription-only medicines in 2013 accounted for € 445 million, representing 14% of the total health expenditure (2,3).

To control public pharmaceutical expenditure, Slovenia uses different cost-containment measures. As in most European countries, the pricing of reimbursed medicines in Slovenia is based on the external reference pricing system (1), defined by the Rules on Determining the Prices of Medicinal Products for Human Use (4). The maximum allowed price (MAP) of generics is determined on the basis of the average price in the three reference countries (Austria, France, and Germany), whereas the MAP of originators and biosimilars is determined on the basis of the lowest price among the reference countries (5). However, MAPs are wholesale prices that usually represent the theoretical prices and are typically not the final or actual prices reimbursed by the public health care payer (6). This is why, for medicine cost control, additional pricing and reimbursement procedures are usually carried out, such as public payer action of setting the maximum reimbursement price (MRP). Slovenia is, thus, one of at least 19 European countries that regulate the (generic) medicine market by using the generic reference pricing (GRP), in which the reference price or MRP is defined for a group of interchangeable (bioequivalent) medicines (7-9).

In Slovenia, the GRP is controlled by the Health Insurance Institute of Slovenia (HIIS), the sole public health care payer in the country, and it has been in use since November 2003. The list of interchangeable (bioequivalent) medicines is determined according to the Medicinal Products Act (10) and Rules on Conditions and Procedure for Determining Interchangeable Medicinal Products (11) by the Agency for Medicinal Products and Medical Devices of the Republic of Slovenia (12). The MRP for a group of interchangeable medicines is determined by the HIIS and based on the lowest wholesale medicine price within the group; however, the condition of at least 0.5% market share of the reference medicine must be met. MRP is (re)set every 2 months, followed by a 2-week transition period, during which the medicine prices under the GRP have to be adjusted to the changes in the MRP values. However, manufacturers are not obligated to adjust the medicine price. In addition, they may adjust the wholesale medicine price at the wholesalers without having to officially communicate it to the HIIS. Nevertheless, if the final actual wholesale medicine price exceeds the MRP, co-payment is required from the patients (7,13). The retail price of medicines under the GRP reimbursed by the HIIS consists of a MRP (wholesale level), community pharmacy dispensing fee, and 9.5% value added tax (VAT) (Figure 1).

**Figure 1:**
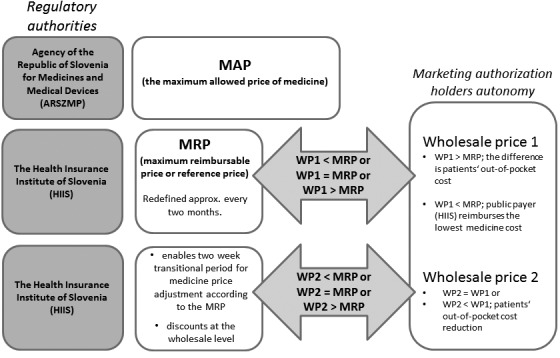
Pricing procedures and price types of the medicines included in the generic reference pricing system in Slovenia. The relation between regulatory authorities and marketing authorization holders involved in pricing of medicines included in the generic reference pricing. Wholesale prices (WP)>MRP: medicines with wholesale price higher than maximum reimbursable price; WP<MRP and WP = MRP: medicines with wholesale price lower and equal to the maximum reimbursable price.

The effect of the GRP as a cost containment measure was already assessed in Italy (14), Portugal (15), Spain (16), and British Columbia, Canada (17). The trends in medicine prices under reference pricing were analyzed in Germany, Spain, and France (9). Also, two studies evaluated the GRP introduction into the Slovenian health care system (18,19). The question remains about the effect of the GRP system on medicine prices in the countries that reference Slovenia within the external reference pricing if the actual prices resulting from the GRP implementation are used instead of the currently used official MAPs (18,20). Furthermore, the manufacturer activities leading to medicine prices adjustments to reach or approximate the maximum reimbursable level are not always clear (21,22). Therefore, aside from being a cost-containment measure, the GRP could also be a factor decreasing the transparency of actual medicine prices in a given country. Furthermore, it has been indicated that the GRP generates savings in the short term; however, there is scarce evidence on its long term effects and the reduction of MRP (18,23-25). In addition, similarly to other countries, in Slovenia GRP implementation was followed with the therapeutic reference pricing system (TRP) system introduction at the end of 2013.

The aim of the study was to assess the impact of the GRP system on prices and cost of medicines approximately 8 years after its introduction and before the implementation of the TRP system. Additionally, the study aimed to assess out-of-pocket cost for patients taking medicines included in the GRP.

## Material and methods

### Methods

**Figure 2:**
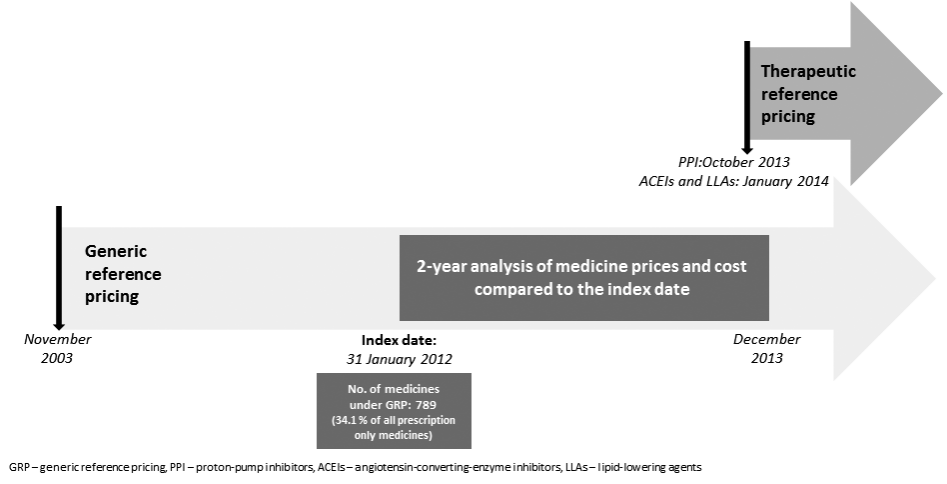
Duration of the generic reference pricing system and therapeutic reference pricing system implementation in Slovenia and placement of the 2-year study period. The index date is a reference time point used for comparisons of medicine prices and cost.

The retrospective study included all prescription-only medicines used in outpatient care (N = 789) covered by the GRP system on January 31, 2012 (Figure 2). Overall, they accounted for 34.1% of all reimbursed prescription-only medicines. Medicine prices and cost were analyzed in the period from January 31, 2012 to December 31, 2013 after every update of the MRPs (N = 11) and were compared to the prices and cost on January 31, 2012 (index date).

### Data sources

Three wholesale prices of medicines were obtained for the purpose of the analysis. The MAP of medicines was obtained from a publication issued by the Agency for Medicinal Products and Medical Devices of the Republic of Slovenia. MAP of medicines could be adjusted twice a year based on the marketing authorization holder (MAH) application, whereas the list of MAPs is refreshed almost every month. However, MAP data from the last publication before each update of the MRP were used for the analysis (26). As the HIIS only announces the MRP of the medicines and does not publish data on the actual medicine prices (27), data on the actual wholesale medicine price within and after the transitional period, MRP, and co-payments without VAT for the medicines included in the GRP were obtained from the lists of actual wholesale prices. These lists are prepared by the main wholesaler in Slovenia in support of pharmacy operation and they are not publically available. Furthermore, the health claims data on outpatient prescription-only medicines obtained from the HIIS were used to define the medicine consumption for every MRP update period. The information on whether a medicine is an originator or generic brand was also included in the final data set.

### Data analysis

Descriptive statistical methods were used to assess the time trends of medicine prices, the budget impact of the GRP system, and medicine price adjustments to the changes in the maximum reimbursable level. Analyses were performed in Microsoft Excel 2010 and IBM SPSS v. 24 (license holder: University of Ljubljana, Faculty of Pharmacy) (28,29). Descriptions of individual parameters defined for each of the 11 MRP update periods are given below.

*Time trends of medicine prices.* To analyze the effect of the GRP system, the time trends of medicine prices were graphically analyzed for each price type, including the MAP, MRP, and actual wholesale price. The average for each price type was determined for all medicines together and separately for the generic and originator medicines.

*Budget impact of the GRP from the public payer perspective*. In order to assess the budget impact of the GRP from the public payer perspective, the medicine cost based on the MAP and medicine cost based on the MRP were determined for each maximum reimbursable level update period. Data on medicine prices and consumption were taken into account. Assuming that without the GRP, the MAP of medicines would be reimbursed by the public payer, the maximum possible budget impact of the GRP was determined.

Additional cost savings for the HIIS resulting from the medicines being priced below the MRP were also assessed. First, the percentages of medicines priced below the reimbursable level during the transitional period and afterward were determined. Next, additional cost savings were determined by taking into account the difference between the actual wholesale price and the MRP, and the consumption data for each medicine priced below the reimbursable price.

*Medicine price adjustments at the wholesale level during the transitional period*. The cost savings and cost increases caused by the wholesale price adjustments were assessed by taking into account the actual wholesale prices at the beginning of the transitional period, discounts or price increases made by the manufacturers, and the medicine consumption (Figure 1). The analysis was performed separately for the medicines with actual wholesale prices equal to MRPs, separately for those with actual wholesale prices lower than MRPs, and separately for those with actual wholesale prices higher than MRPs in order to determine whether price adjustments were actually more frequent for the medicines priced above the maximum reimbursable level, as would be expected.

*Patients’ out-of-pocket cost*. The actual total patients’ out-of-pocket cost was determined. In addition, potential cost savings for the patients resulting from discounts given by the manufacturers at the wholesale level were determined as the difference between the potential medicine cost that would occur without the price reductions at the wholesale level and actual co-payments. However, if wholesale-level discounts were not offered, medicine requiring co-payment could be replaced by the cheaper medicine without co-payment, and potential cost savings would be lost. The assessment of the actual total patient out-of-pocket cost and potential savings did not include 9.5% VAT.

## Results

### Time trends of medicine prices

**Figure 3:**
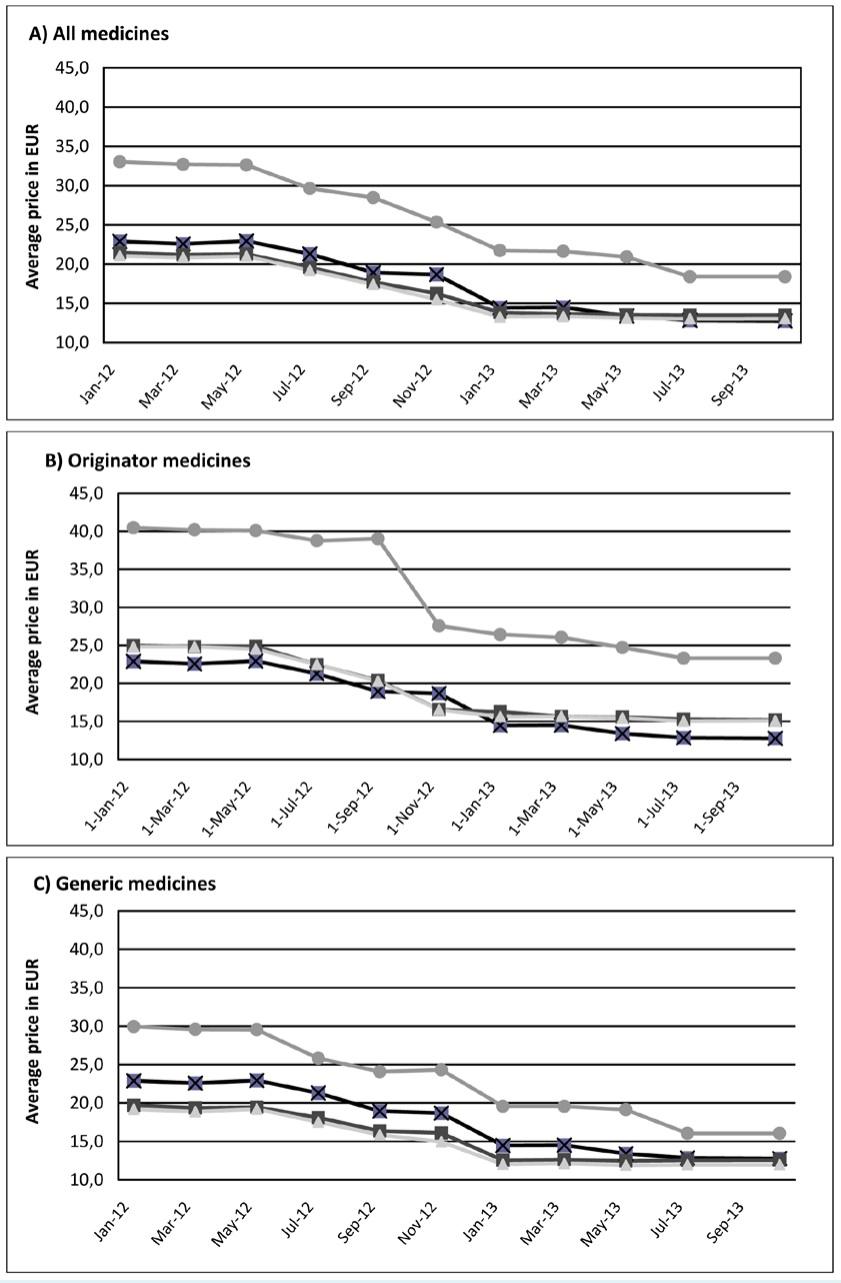
Time trends of the medicines' average maximum allowed price (circles), average maximum reimbursable price (crosses), average wholesale price in the transitional period (squares), and average wholesale price after the transitional period (triangles) within the study period: (**A**) all medicines; (**B**) originator medicines; (**C**) generic medicines.

The average MAP and average MRP for all medicines decreased by approximately 45% within the 2-year study period (Figure 3A). The average actual wholesale prices decreased by approximately 35%. The average MAP was approximately 30% higher than the average MRP, ranging between 28% and 35%, depending on the maximum reimbursable level update.

The average MAPs of originators and generics decreased comparably: by 42% and 46%, respectively (Figure 3B and 3C). The average MAP of originators considerably decreased, by almost 30% at the end of 2012. The average MAP was 35%-50% higher than the average MRP for the originators and 20%-30% higher for generics. The actual wholesale prices of originators were on average 10%-15% higher than the MRP. The exception was the last period in 2012 (the sixth period), when the average actual wholesale price of originators was actually lower than the average MRP. In contrast, the average actual wholesale price of generics was approximately 15% below the average MRP. However, in the last three updates of the MRP, the average actual wholesale price of generics was nearly the same as the average MRP.

### Budget impact of the GRP from the public payer perspective

The maximum possible budget impact of the GRP system, shown as medicine cost reduction, ranged from 29.7 to 34.4%, depending on the MRP update period (Table 1). While the GRP system impact on cost reduction of original medicines did not differ considerably between the 11 maximum reimbursable price update periods, for generic medicines a downward trend was observed. The additional cost savings for the HIIS resulting from medicines being priced below the MRP, shown as medicine cost reduction determined based on their MRP, were 0.04%-3.10%.

**Table 1:** Budget impact of the generic reference pricing (GRP) from the public payer perspective for all medicines, for generics and originators, and additional savings for the public payer due to medicines priced below the maximum reimbursable price (MRP)

Budget impact of the GRP											
Date of the MRP update	31-Jan-12	29-Mar-12	31-May-12	31-Jul-12	28-Sep-12	30-Nov-12	31-Jan-13	27-Mar-13	29-May-13	31-Jul-13	1-Oct-13*
All medicines											
Medicine cost based on MAP (€)	28,824,572	28,291,799	27,239,198	25,813,256	26,876,991	25,885,304	21,541,174	24,391,289	23,420,158	21,983,709	22,389,126
Medicine cost based on MRP (€)	19,194,295	18,547,091	17,992,912	17,089,494	17,923,536	17,219,534	14,792,874	16,549,583	16,467,767	15,293,061	15,302,243
Cost reduction due to the GRP (%)	33.41	34.44	33.94	33.80	33.31	33.48	31.33	32.15	29.69	30.43	31.65
Originators											
Medicine cost based on MAP (€)	15,222,058	15,225,757	14,467,390	13,882,630	14,527,980	13,798,243	11,691,347	13,410,831	13,414,991	12,359,944	12,610,356
Medicine cost based on MRP (€)	8,879,346	8,506,672	8,212,984	7,783,323	7,953,938	7,597,313	6,510,357	7,361,122	7,363,945	6,799,743	6,837,633
Cost reduction due to the GRP (%)	41.67	44.13	43.23	43.93	45.25	44.94	44.31	45.11	45.11	44.99	45.78
Generics											
Medicine cost based on MAP (€)	13,602,515	13,066,042	12,771,809	11,930,627	12,349,011	12,087,060	9,849,827	10,980,459	10,005,168	9,623,765	9,778,771
Medicine cost based on MRP (€)	10,314,949	10,040,418	9,779,928	9,306,171	9,969,598	9,622,221	8,282,516	9,188,462	9,103,822	8,493,318	8,464,610
Cost reduction due to the GRP (%)	24.17	23.16	23.43	22.00	19.27	20.39	15.91	16.32	9.01	11.75	13.44

Additional savings due to medicines priced below the MRP											
Proportion of medicines with wholesale price below the MRP (%)											
In transitional period	8.59	3.00	2.58	17.00	1.72	2.99	2.88	1.91	11.73	8.40	3.09
After transitional period	10.72	2.96	3.94	15.18	1.74	9.95	2.88	1.90	14.81	5.32	3.09
Additional medicine cost savings (€)	282,496	42,552	39,610	146,640	6,714	175,684	46,189	50,618	510,634	69,416	94,312
Cost reduction due to medicines priced below the MRP (%)	1.47	0.23	0.22	0.86	0.04	1.02	0.31	0.31	3.10	0.45	0.41

*MAP – maximum allowed price.

†In order to compare medicine costs between maximum reimbursable level updates, for a period with a start date of October 1, a hypothetical 2-month period was taken into account when determining medicine cost. In reality this period was extended due to the therapeutic reference pricing system implementation in the beginning of 2014. In contrast, additional cost savings are presented for the whole period (3 months).

### Medicine price adjustments at the wholesale level

**Figure 4:**
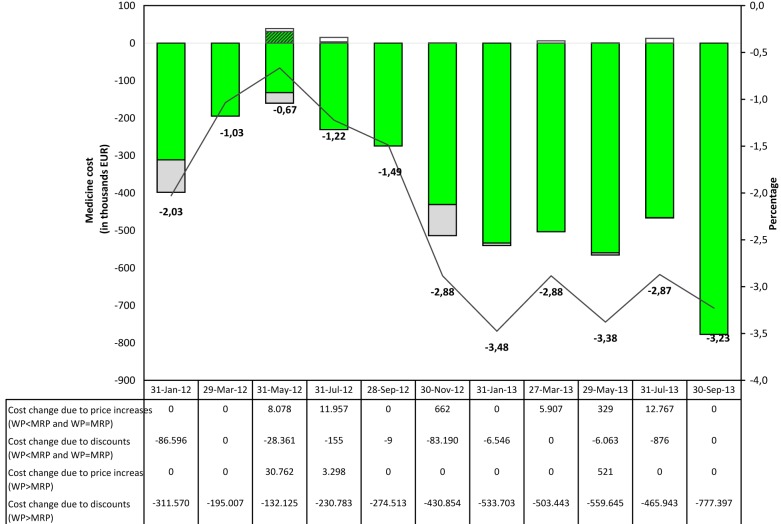
Cost savings, cost increases, and net effect of medicine price adjustments at the wholesale level for each maximum reimbursable price update period. Cost change due to price increases for medicines with wholesale price lower and equal to the maximum reimbursable price (WP<MRP or WP = MRP, white bars), cost changes due to price increases for medicines with WP higher than the maximum reimbursable price (WP>MRP, striped bars), cost change due to price discounts at the wholesale level for medicines with WP lower and equal to the maximum reimbursable price (gray bars), and cost changes due to price discounts at the wholesale level for medicines with WP higher than the maximum reimbursable level (dotted bars). Net effect (black line) of price adjustments at the wholesale level is determined in relation to the pharmaceutical expenditure considering WP without price adjustments at the wholesale level.

At the wholesale level, medicine price adjustments were mostly price reductions. However, a price increase after the transitional period was detected for some medicines in six of eleven periods. Medicine price reductions were most common for medicines that had an actual wholesale price higher than the MRP (Figure 4). Cost reductions resulting from the discounts for the medicines with the actual price higher than MRP varied from approximately € 130 000 to € 780 000, depending on the maximum reimbursable level update period. For medicines with the actual price equal to and lower than MRP price, reductions were rare. However, the net effect of price adjustments at wholesale level varied from 0.7%-3.5%, depending on maximum reimbursable level update period.

### Patients' out-of-pocket cost for the medicines included in the GRP

**Figure 5:**
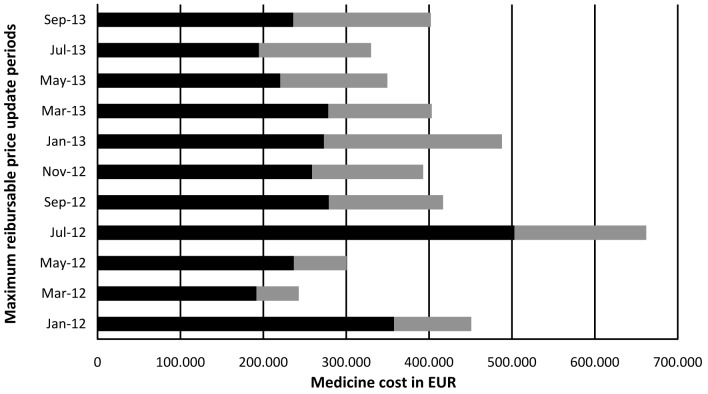
Patients’ out-of-pocket costs for the medicines included in the generic reference pricing system (black bars) and potential cost savings for patients due to discounts offered by the marketing authorization holders at the wholesale level (gray bars).

Manufacturers contribute to patient cost savings with discounts given on the day of the MRP update (at the beginning of the transitional period) or with new or additional discounts given at the end of the transitional period. Therefore, the percentage of medicines requiring co-payment after the transitional period was lower than during the transitional period for most of the MRP update periods (Figure 5). The exception was the third period (May 2012), when the percentage of medicines requiring co-payment was the same during and after the transitional period. The percentage of medicines requiring co-payment differed between the maximum reimbursable level update periods, varying between 9% and 16%. The exeption was the fourth period (July 2012), when the percentage of medicines requiring co-payment was 33% during the transitional period and 19% afterward. The average difference between the percentage of medicines requiring co-payment during the transitional period and afterward was 3.5 percentage points. Furthermore, the proportion of patients who co-paid for any medicine included in the study was on average 13%, varying from 4.6% in the second period (March 2012) to 39.1% during the transitional period of the fourth period (July 2012).

## Discussion

We found a downward trend in all types of prices of medicines included in the GRP system. Average MAP and average MRP decreased by approximately 45%, while the actual wholesale prices decreased by approximately 37%. Moreover, the average actual wholesale price was approximately 35% lower than the average MAP. The MAP decline could be a consequence of price decreases in three reference countries, ie, Germany, Austria, and France. However, it could also be a consequence of the frequent changes in external reference pricing methodology introduced by the changes of the Rules on Determining the Prices of Medicinal Products for Human Use (5). In contrast, the MRP and actual wholesale prices depend on the local generic competition and activities of the HIIS, which changes (reduces) MRPs of interchangeable medicines approximately every two months and negotiates prices with MAHs for the reimbursement of medicines. Slovenia is one of the 25 European countries using discounts and rebates granted by the pharmacy industry as an additional measure when price regulation does not lead to the desired results (21). For the 19 countries referencing Slovenia within the external reference pricing system, it means that they have risked overpaying as prices are compared on the basis of official prices instead of the actual prices paid by payers in a given country (30). The medicine prices that were considered for the comparison have most probably influenced medicine prices in Croatia, Serbia, Bosnia and Herzegovina, and Portugal, which use external reference pricing as the main pricing criterion, apply average as price calculation, and have only two more countries beside Slovenia in the reference country basket. In addition to Slovenia, Croatia references Italy and Czech Republic; Serbia references Croatia and Italy; Bosnia and Herzegovina references Croatia and Serbia; and Portugal references Spain and France (31). However, like other European countries, these countries also use additional cost-containment measures to achieve the acceptable price level. In this way, the effect of external reference pricing is undermined, as non-stationary and confidential price cuts cannot be transferred to the reference countries (32). Hence, a better effect of external reference pricing system as a cost containment measure would be generated if the price actually paid by public payers was referenced (30).

Studies evaluating the performance of the GRP in Germany, Italy, and Norway reached a similar conclusion, ie, that the GRP was generally associated with a drop in medicine prices (14,23,33,34). For example, after the GRP introduction in Sweden, medicine prices decreased by about 15% from 2003 to 2005, while market prices for generic medicines decreased by approximately 40% (35,36). In addition, one study showed a significant price decrease of selected medicines after the GRP introduction in Slovenia, compared to the period before GRP introduction (18). Another study evaluating the impact of medicine pricing regulation on the public pharmaceutical expenditure in Slovenia also confirmed a price decrease after the GRP introduction (19). Furthermore, our study also found that the GRP as a reimbursement policy in Slovenia was effective in the long term. Beside the repeated and persistent MRP reduction induced by the HIIS, prices largely underwent adjustments due to the changes in the MRP values.

Price reduction trends were similar for generic and originator medicines. The greatest decrease in MAP of originator medicines occurred at the end of 2012. There was no change in medicine pricing regulation that could explain this significant price drop, but the official price of originator medicines in one of the three reference countries significantly decreased. Further, we found a greater difference between MAP and actual wholesale prices for originators compared with generics, and the average wholesale price of originators was approximately 20% higher than that of generics. The study by von der Schulenburg et al (37) refuted the concern that originator medicines did not respond to generic competition or price regulation based on the GRP system post-patent expiry, due to the originator manufacturers’ loss of interest in keeping market share by lowering prices after the entry of generics. Similarly, the present study showed that originator prices may indeed decrease as a result of the GRP system. We confirmed that the reference pricing system caused an obvious and almost compulsory reduction in the price of all medicines subject to this system to a varying degree in different periods, with a greater reduction for originators than for generics (9).

From the public payer perspective, the maximum possible budget impact of the GRP was a cost reduction of medicines included in the system of approximately 30%. This represents approximately € 42 million annual medicine cost savings, which is approximately 10% of the total outpatient prescription-only medicine expenditure in Slovenia. Furthermore, the total cost of medicines included in the GRP system based on the MRP decreased by approximately 20%. These findings confirm a persistent effect of the GRP as a cost containment measure. Although the HIIS reduced medicine expenditures by approximately € 4 million due to lowering MRPs of medicines, the total amount of patient co-payments was approximately € 3 million. This means that the HIIS benefited from the GRP system more than the patients taking medicines included in this system. However, MAHs were willing to lower the medicine prices to a certain extent and thus contributed to the reduction of patient out-of-pocket cost.

Taking price and cost reduction of medicines included in the GRP system into account, the impact of the GRP on cost reduction was approximately half of the medicine price decrease. The reason could be that the MRP of the most used medicines did not decrease the most, as the overall medicine consumption did not vary significantly between the periods of the maximum reimbursable level update periods. The medicines with the highest consumption were cardiovascular system medicines, followed by alimentary tract medicines and metabolism and nervous system medicines, and their MRP decreased by 23%, 24%, and 38%, respectively. Like in other countries, only a small proportion of medicines was priced below the maximum reimbursable level. Thus, aside from stimulating price competition and lowering medicine prices, the GRP system constitutes a barrier to further price competition, because price reductions beyond those imposed by regulations are very rare (9,13,38).

Non-transparency of actual medicine prices in Slovenia, including price adjustments at the wholesale level as an alternative to the official price reduction agreements with the HIIS, has often been considered a disadvantage of the Slovenian pricing system (39). However, we showed that medicine price adjustments at the wholesale level had a marginal impact on the total pharmaceutical expenditure, as they decreased expenditure of medicines included in the GRP by 0.7%-3.5%. Furthermore, most of them were made for medicines priced above the maximum reimbursable level, indicating that discounts at the wholesale level may have a significant impact on patients’ out-of-pocket costs. However, price reductions could also be inferred as a factor enabling manufacturers to obtain the desired pharmaceutical market share. This is why when co-payment is required, substitution with a reference medicine without co-payment is more likely (23).

Although the GRP system introduction in Slovenia was initially received with skepticism, it enhanced price competition, giving manufacturers an incentive to reduce prices to have their products reimbursed by the public payer and to reduce patients’ out-of-pocket cost (14,37). Also, concerns regarding the withdrawal of products from the Slovenian market or MAHs leaving the Slovenian market, and the associated decrease in patient access to medicines in Slovenia, were not realized. On the contrary, savings achieved due to the GRP system implementation should contribute to better patients’ access to both the existing and the new and expensive medicines.

Most studies evaluated the impact of the GRP as a cost-containment measure by comparing the prices of medicines before and after its introduction (9). According to our results, the GRP was an effective cost-containment measure, not only immediately after its introduction, but also in the long term. Still, the persistent price impact of the GRP is generally unclear, but evidence from Spain (16,40,41), Germany (42), Belgium (43), Italy (14), and British Columbia, Canada (17) shows a medium- to long-term decrease in MRPs of medicines under the GRP. We also analyzed if the potential inability of the GRP system to further reduce MRPs was one of the reasons for the introduction of TRP in Slovenia. Our results indicated that the TRP was aimed to provide additional savings needed for the sustainability of the Slovenian health care system and to decrease the differences in medicine prices between the therapeutically equivalent medicines rather than only bioequivalent medicines.

We assumed that the GRP system was the main pricing regulation system contributing to the medicine price and cost decrease for the medicines included in this system, despite some other reimbursement regulations in Slovenia. Most medicines included in the GRP system had a price equal or close to the maximum reimbursable level; consequently, the GRP could be inferred as a factor contributing the most to the reduced price of these medicines. Further, only medicines included in the GRP system were considered, and there is a lack of data on the actual wholesale prices of other medicines that could be used for comparison.

There are different factors contributing to the price decrease to the MRP. Our focus was on the price adjustments at the wholesale level as there are no data on manufacturers’ activities that may contribute to price decrease to the maximum reimbursable level. In this regard, we used the lists of actual wholesale medicine prices and wholesale-level price adjustments (discounts or price increases) that are commonly valid at the national level. As wholesale medicine prices were used in the analysis, community pharmacy dispensing fee and VAT were not taken into account.

In conclusion, the long-term performance of the GRP system was associated with an approximate 45% decrease in the MRP, or an approximate 20% cost reduction, in the 2-year period, 8 years after the GRP system introduction and before the TRP system introduction. Moreover, all types of prices for medicines included in the GRP system had a similar downward trend. The medicine price adjustments at the wholesale level were particularly important from the patients’ point of view. The GRP seemed to be an effective cost-containment measure not just immediately after its introduction, but also in the long term.
